# Supplementary material for: Smart Packaging with Disposable NFC-enabled Wireless Gas Sensors for Monitoring Food Spoilage
Source: ACS Sens. 2024 Dec 16;9(12):6789–99. doi: 10.1021/acssensors.4c02510 (PMC11686504; doi:10.1021/acssensors.4c02510)
Supplement: Supplementary file 2 — se4c02510_si_002.pdf [file se4c02510_si_002.pdf]

Smart Packaging with Disposable NFC-enabled Wireless Gas Sensors for Monitoring Food Spoilage

Atharv Naik[1], *Hong Seok Lee*[1], *Jack Herrington*[1], *Giandrin Barandun*[1,2], *Genevieve Flock*[3], *Firat Güder*[1,4]* *and Laura Gonzalez-Macia*[1]*

[1] Department of Bioengineering, Imperial College London, London SW7 2AZ, United Kingdom

[2] BlakBear Ltd, 185 Tower Bridge Rd, London SE1 2UF, United Kingdom

[3] Combat Capabilities Development Command Soldier Center, Natick, MA 01760, United States

[4] Bezos Centre for Sustainable Protein, Imperial College London, London SW7 2AZ, United Kingdom

*Corresponding Authors: guder@imperial.ac.uk, m.gonzalez-macia@imperial.ac.uk

Table S1. Material specifications for the membranes used to encapsulate PEGS.

Commercial name	Short name	Material	Thickness (mm)	Biocompatibility
POREX Porous PTFE Medical Materials MD25	MD25	PTFE	0.19	Use for medical applications [1]
Nitto TEMISH porous PTFE S-NTF8031J	TEMISH	PTFE	0.13	Applications: medical face masks, respirators, air purifiers [2]
3M™ Medical Film 9832F, Polyurethane	PU	Polyurethane	0.02	Suitable for wound care dressings and wearable devices [3]
Biaxially oriented polyester (OPET) film (OCLF)	mPET	PET	0.0127	Food packaging industry [4]
Cellulose-based compostable sealing film	Cellulose	Cellulose	--	Food packaging industry [4]
Polyurethane-based tattoo film	Tattoo	Polyurethane	--	Tattoo Wrap Waterproof Wound Antibacterial Transparent Bandage [5]

[1]MD25 – POREX Virtek PTFE Hydrophobic Medical Venting Porous Membrane Sheets, https://www.porex.com/product/porex-virtek-ptfe-hydrophobic-medical-venting-porous-membrane-sheets-md25/; [2] https://www.nitto.com/eu/en/products/temish_search/about/; [3]https://www.3m.co.uk/3M/en_GB/p/d/v000266868/; [4]Bullseye Food Packaging https://www.bfpuk.com/; 5 Patrick F. Mcclernon, Russell Blette, Donna Dearinger, *Transparent breathable polyurethane film for tattoo aftercare and method*, WO 2010/042511 A1.

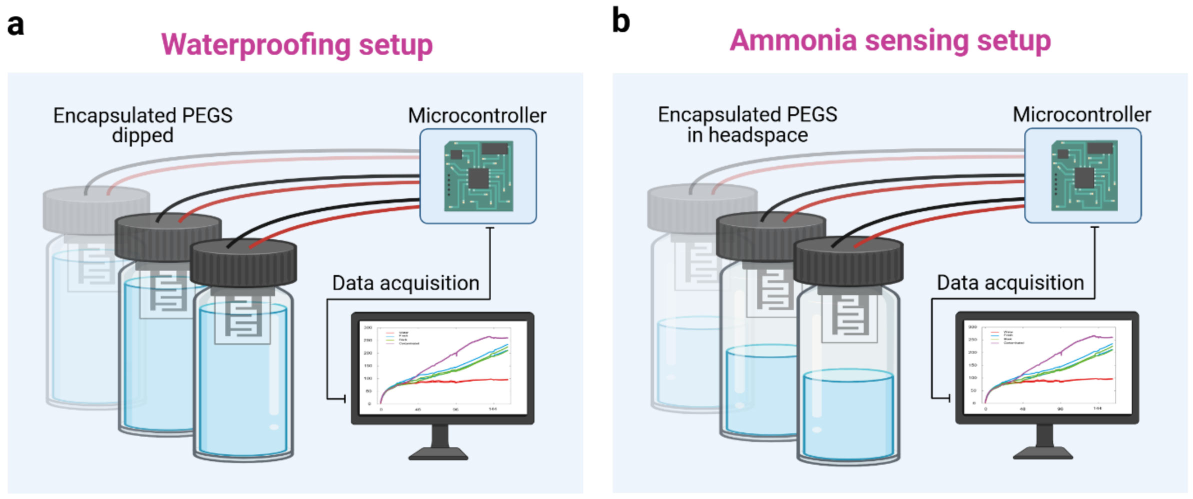

Figure S1. **a** Scheme of experimental setup used to evaluate the waterproofing properties of the encapsulation membranes (PEGS dipped into DI water). **b** Scheme of experimental setup used to measure changes in conductance over time of encapsulated PEGS placed in the headspace of a vial containing 1 mM NH_4OH solution and comparison to non-encapsulated PEGS response.

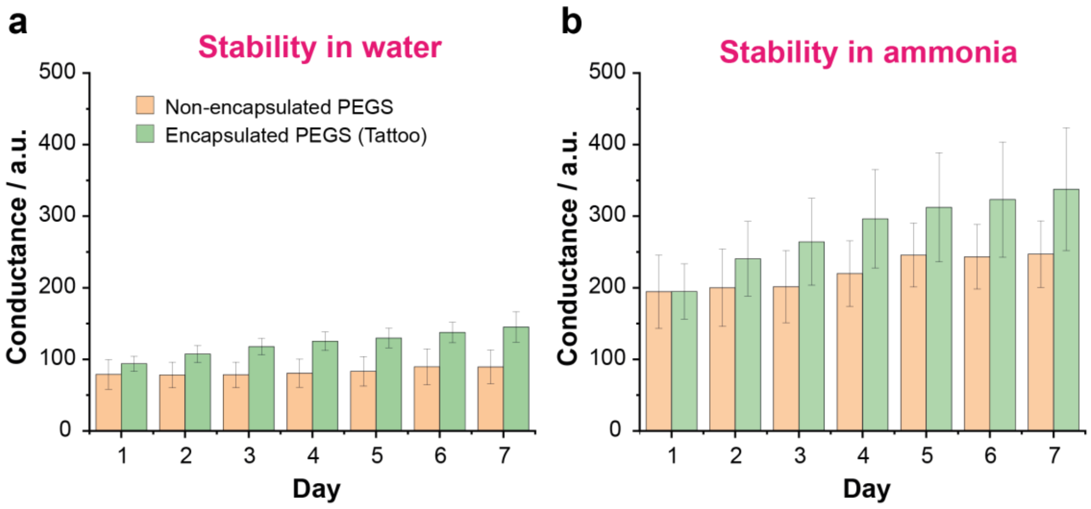

Figure S2. Stability study of non-encapsulated PEGS and PEGS encapsulated with polyurethane-based tattoo film. Sensors were continuously exposed to: **a** water, and **b** 1 mM NH_4OH and the conductance changes were recorded for one week (n = 4).

Table S2. Breakdown of the cost for the fabrication of the NFC-enabled system integrated with encapsulated PEGS (Tattoo film) for the monitoring of spoilage in spinach

Component	Price per unit (USD)
PEGS	0.02
3M 9703 Conductive Tape	0.06
SIC4341 Chip	0.01
Passive components (capacitors)	0.01
Flexible PCB	0.16
Tattoo film	0.08
**TOTAL (USD)**	**0.35**

Supplemental video. Video showing the procedure for the monitoring of spoilage in bagged spinach using the NFC-enabled system integrated with encapsulated PEGS (Tattoo film). A user-friendly version app was used to communicate the level of spinach freshness by “Fresh” or “Not Fresh” messages.
